# First report of the agricultural biocontrol agent *Bacillus velezensis* and foodborne outbreak due to rope spoilage in cakes

**DOI:** 10.1128/aem.02570-24

**Published:** 2025-03-28

**Authors:** Donald E. Brannen, Jere Marks, Richard Schairbaum, Rick Bokanyi

**Affiliations:** 1 Greene County Public Health, Greene County 46534 , Xenia, Ohio, USA; 2 Ohio Department of Health Laboratory, Reynoldsburg, Ohio, USA; Anses, Maisons-Alfort Laboratory for Food Safety, Maisons-Alfort, France

**Keywords:** *Bacillus*, *Bacillus velezensis*, food quality, outbreak

## Abstract

This paper presents an outbreak involving retail bakery cakes and a celebration at a business with multiple sites. *Bacillus* spore-forming bacteria have been identified as the cause of “ropey bread” spoilage for over 100 years. Causative strains of rope spoilage include *B. subtilis* and other *Bacillus* species. Thirty-five employees identified as having been at a celebration event across 11 sites were questioned to characterize the amount of cake eaten, observations, and symptoms. No human specimens were obtained, but leftover cake was analyzed by the Ohio Department of Health Bureau of Public Health Laboratory. The odds that cake caused symptoms were 9.23 (1.02–83). The presence of odor decreased the amount eaten by 151 g (103–205 g), *P* = 0.001. Enteric symptoms developed 0.41–4.5 hours after exposure, with 5 of 12 cases having latent diarrhea. Cakes were positive for *Bacillus velezensis*. This appears to be the first mention of *B. velezensis* as a contributor to the re-emergence of rope spoilage in the bakery industry. Updates to the *Bacillus* taxonomy starting in 1973 may account for why *B. subtilis* and other *Bacillus* strains are historically listed without mention of *B. velezensis* as a cause of rope spoilage. Given the ability of *B. velezensis* to be an effective biocontrol agent and to grow at pH and water activity levels like many baked goods, there is a need to study issues along the entire food chain to balance the impact on biocontrol additives on food production and safety.

**IMPORTANCE:**

This appears to be the first mention of *Bacillus velezensis* as a contributor to the re-emergence of rope spoilage in the bakery industry. Given the ability of *B. velezensis* to be an effective biocontrol agent and to grow at pH and water activity levels like many baked goods, there is a need to study issues along the entire food chain to balance the impact on biocontrol additives on food production and safety.

## INTRODUCTION

Are there unintended consequences in the shift in the agricultural industry from using chemicals to natural substances as biocontrol agents? This paper will present an analysis of an outbreak involving a retail bakery and a business-related celebration that prompted this question. This background will discuss the celebratory event, the bakery, and the causative agent (a bacterium from the *Bacillus* genus). The celebration occurred on 12 May.

The genus *Bacillus* is an aerobic, sporulating, rod-shaped bacteria that are ubiquitous in nature (1). Public health authorities have a heightened focus on disease-causing species of *Bacillus* like *B. anthracis,* the causative agent of anthrax, and *B. cereus* for its potential in foodborne outbreaks, such as when foods are left out unrefrigerated or undercooked (2, 3). In the United States from 2009 through 2021, foodborne outbreaks involving *B. cereus* caused over 4,900 illnesses (4). While implicated food vehicles in *B. cereus* foodborne outbreaks are varied; beans, rice, or pasta were associated in over 40% of those outbreaks (4, 5). If a potential source of foodborne outbreak was from a grain-based food, *B. cereus* would be a suspect causative agent.

**Fig 1:**
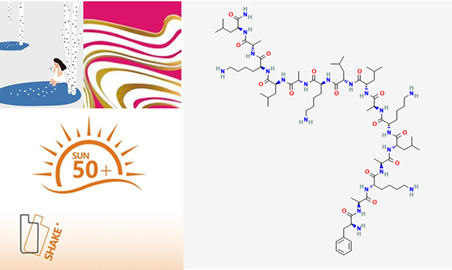
Lipopeptides are used widely in industry and pharmaceutical applications, including antibiotics, fungal inhibitors, cosmetics, and other uses. For example, oligopeptide-10 is used as an eye lash enhancer and in sunscreen. Product label images and chemical structure from the National Center for Biotechnology Information, National Library of Medicine (NLM), accessed 12 June 2024.

**Fig 2:**
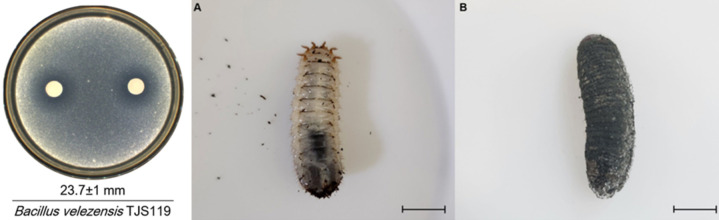
*B. velezensis* inhibits insect (>94%) and plant pathogens. Agar plate showing inhibition of fungus, with healthy and fungal-infected insect larvae. (Republished from *Frontiers in Microbiology* [14].)

*Bacillus velezensis* was isolated from the Velez River in Spain in 2005 (6). *B. velezensis* are Gram-positive with optimal growth conditions at a pH of 7.0 to 8.5 (range 5 to 10) and an optimal temperature of 30°C–35°C (86°F–95°F) (7). Secondary metabolites are small biologically active molecules produced by many lifeforms that are not essential for growth or reproduction (8). These metabolites can have a wide range of effects and may produce a competitive advantage. *B. velezensis* produces phosphate, pectinase, chitinase, protease, indole-3-acetic acid, and hydrogen cyanide as secondary metabolites making them potential biocontrol agents (7). Lipopeptides biosurfactants are composed of a lipid tail linked to a short linear or cyclic oligopeptide (9, 10). Lipopeptide biosurfactants produced by *Bacillus* species have received attention for their diverse environmental and pharmaceutical applications (11). *Pseudomonas* and *Bacillus* species develop biofilms that use lipopeptides in their construction. Lipopeptides are important in polymyxin antimicrobials (there have been rising resistance to these antibiotics in Southern China from the gene mcr-1 in bacterial plasmids in *Enterobacteriaceae*) (12). The competitive advantage and use as an antimicrobial is from the lipopeptide interaction with the cell wall lipopolysaccharides that disrupt the competing microbe or infectious agent. An example of an oligopeptide is shown in Fig. 1 (13). Oligopeptide-10 has a molecular form of C_78_H_140_N_20_O_15_. It is used as a lash-enhancing serum, moisturizing sunscreen cream, and other applications. *B. velezensis* is used in agriculture to control insect and wheat blast disease (Fig. 2) (14, 15). *Bacillus* genera belong to the family of bacteria Bacillaceae which produce endospores and are mostly found in the soil. *B. velezensis* is an inhibitor of Fusarium head blight during grain farming. Agricultural products known as inoculants are sold throughout the world to promote wheat growth and prevent blight. These inoculants are microbial biostimulants containing spores of naturally occurring soil bacteria. For example, *B. velezensis* strain FZB 45 is used to stimulate root growth and mobilizes nutrients in the soil to support the availability of plant nutrients, which leads to an increase in yield. These types of products are either mixed with the seeds before planting, mixed with the soil during planting, and/or applied throughout the season to the plant’s base. When applying on the seeds, the microbial biostimulant is mixed to ensure a uniform coating of seeds. The manufacturer’s directions have instructions for specific amounts and application methods. These products are marketed to wheat farmers for root growth enhancement, nutrient uptake, and overall plant health.

As opposed to corporate food retail establishments, small retail bakeries have their own unique challenges related to food safety. Considerations for the operational success of a small retail bakery include maximization of floor space, storage of ingredients, areas needed for preparation, baking, cooling, decorating, packaging, and an area for customer ordering and pickup. Not only does the bakery floor plan have to be designed within health and safety regulations, but compliance inspections must be conducted to assist in preventing foodborne illnesses.

Humans get an average of 48% of their calories from grains (16). Pacher et al. describe ropiness in bread as a “re-emerging spoilage phenomenon” (17). *Bacillus* spore-forming bacteria have been identified as the cause of ropey bread spoilage (18). Bread rope spoilage was characterized over 100 years ago (19). The signs of rope bread spoilage are discoloration, rotting pineapple smell, and thin strings when the bread is pulled apart. The smell is caused by volatile compounds including diacetyl, acetoin, acetaldehyde, and isovaleraldehyde (17). While the historical context of ropey bread spoilage is well known, this paper will clearly demonstrate a dose-response relationship between exposure to *B. velezensis* and the presence of symptoms. Known virulence factors of *B. velezensis* include genes encoding hemolysin A (hlyA), cytolysin (cyl), enterotoxins such as hemolysin BL (Hbl), non-hemolytic enterotoxin (Nhe), and cytotoxin K (CytK), which contribute to its pathogenicity (14).

Pepe et al. in their quest to provide a recommendation for a biocontrol strain in bakery products to control rope spoilage in bread found that the majority of causative strains were those of *B. subtilis* and *B. licheniformis*, adding that “other *Bacillus* species might be able to cause ropiness in bread” (20). Spoilage of bakery products is characterized by low acidity and high water activity (a_w_) in the food product (21). Moisture is the total of bound and free water the product contains, and a_w_ is a measure of the “free water” in the food product. The U.S. Food and Drug Administration (FDA) defines a_w_ as the “ratio between the vapor pressure of the food itself, when in a completely undisturbed balance with the surrounding air media, and the vapor pressure of distilled water under identical conditions.” The a_w_ should be maintained with an acceptable range of activity during storage in order to prevent spoilage. If the a_w_ is less than 0.85 for a finished food product, it is “not subject to the regulations of 21 CFR Parts 108, 113, and 114” because the available moisture is reduced to a point that inhibits bacterial growth.

Initiation of investigation on 13 May 2024, the business reported aberrations in cakes that had been ordered as part of the celebration. The initial report to public health stated no one had become ill, so the local public health epidemiologist did not start an outbreak investigation on that date. By 15 May, out of an abundance of caution, the epidemiologist asked if there was any food left and if so, could it be secured and stored appropriately, and asked for a list of persons thought to be exposed, as well as the invoices for the flour the cake was made from. The local public health inspector followed up with a preliminary inquiry to the bakery. After the results of the preliminary inquiry, a second inquiry was made after a consultation with an Ohio Department of Health (ODH) epidemiologist. By 10:33 a.m. on the morning of 15 May, the ODH Epidemiologist requested that the remaining flour not be used, but by this time it was found that the “cake mix” had all been used for the cake order. The ODH epidemiologist continued to explore the feasibility of testing the cakes and leftover ingredients at the ODH Bureau of Public Health Laboratory or the FDA laboratory. The ODH epidemiologist suggested that a survey of symptoms and illnesses be made of persons potentially exposed. After some inquiries by the local epidemiologist, two subjects were found to be ill by the healthcare provider’s infection preventionist. The local epidemiologist requested a survey be sent out, and the business agreed. Based on two persons having been ill, the outbreak investigation was started at that time on 16 May 2024, at 15:23 hours.

## MATERIALS AND METHODS

A case-control survey was sent out to employees identified by the business as affected or likely exposed, as some cakes were removed before being served. Employees from sites that received cakes as part of the celebration that were eaten were selected to be surveyed. This included those who were exposed (ate cake) and unexposed (did not eat cake) as well as symptomatic and asymptomatic. The questions started after the purpose was stated as “information was being collected to help determine the safety of the ingredients and product processing of the cake served on 12 May 2024.” The questions included:

1. How much cake did you eat (enter number of bites, e.g., 0, 1, 2, 3, …)?
2. If you ate any cake at all, please describe the amount (e.g., 1 forkful is about 28 g or about 1 ounce)?
3. Did you notice any of the following? (check all that apply) Off odor, discoloration, stringiness, other (please specify).
4. Did you have any symptoms, even mild ones? Yes, no.
5. If yes, please describe your symptoms (including the length of time from eating the cake to symptom onset).
6. Symptoms (yes, no): headache, nausea, vomiting, cramps, diarrhea, fever, chills, muscle aches, other. If you have any symptoms, please list the date and time they started.
7. Question asking for personal information if a follow-up was asked for.
8. If you had symptoms, when did you start to feel better? Date time.
9. If you did not have any cake but were present, please feel free to provide any observations about the event.

At the request of the epidemiologist, the manager of the Public Health Food Service Program was asked to review past inspection report findings for the bakery, as well as the reported steps conducted in making the cakes for any indication of contributing factors for bacterial proliferation. From the surveys, the amount of cake eaten and symptoms experienced were tabulated. To answer the question about what spoilage characteristics were protective factors against ingesting spoiled food, the average amount of cake eaten by symptom category was compared using independent t tests. The sample of persons was measured on whether they observed ropes, odor, discoloration, or other signs of spoilage and by the amount of cake eaten. Observed spoilage signs were grouped together as “other” due to their singular occurrence (discoloration, fluffy, very dense-moist, taste off, undercooked-doughy). The category of other symptoms included singular occurrences of headache or heartburn. Descriptive estimates of amounts were converted into grams of cake eaten. Bootstrapping was used to provide a robust estimate of the effect of the spoilage warning signs noticed by the person on the amount of cake eaten in grams. Specifically, linear regression with bootstrapping was used to estimate the effect of observable signs of spoilage (ropes, odor, and other signs) on the amount of cake eaten. Regression analysis of the dose in grams of cake eaten among the cases (unexposed subjects were not symptomatic, and many may have avoided eating cake if spoilage was observed) was used to determine the impact on symptoms. A *post hoc* fault modes effect analysis (FMEA) was used to identify potential retrospective hazards related to the agricultural production of the grain, transportation of the grain, the bacteria identified, the bakery’s process, and the transportation and storage of the cakes. Though the FMEA method is not typically used for foodborne outbreaks, it was used as an adjunct method because cakes are typically not considered time-temperature-control for safety (TCS) foods, and a trace back of the lot numbers of the cake mix was not done.

One subject who had no exposure and flu-like symptoms was excluded from modeling but was included in the calculation of odds of illness if exposed. The Fisher’s exact method was used to calculate the odds ratio due to fewer than five observations in some of the cells. Five samples of leftover cakes were sent to the state laboratory for testing. Identification was done utilizing VitekMS matrix-assisted laser desorption/ionization time-of-flight (MALDI-TOF) mass spectrometry (BioMerieux, Cambridge, MA). Supplemental tests were not performed to confirm this identification. The authors realize that in foodborne outbreaks, it is preferred that isolates from human cases are also obtained. In this outbreak, the symptoms were consistent with the symptoms of spore-forming bacteria. Across the multiple sites spread across adjacent cities, ingestion of the food product was the common association among those who were ill and that their symptoms were correlated to signs of rope spoilage. Furthermore, the type of spoilage observed is consistent with *B. velezensis*. Updates to the taxonomy since 1973 have split *B. subtilis* into four conspecific strains: *B. velezensis*, *B. amyloliquefaciens*, *B. subtilis*, and *B. siamensis*. These strains can be evaluated using mass spectrometry that analyzes the fatty acid composition of the bacterial cell membrane, which can vary between species and strains (22). Molecular identification was not done to identify the agent found in the product. The agent was identified using mass spectrometry. For this outbreak, the lab was asked to test the cake for *B. cereus*. The lab ran it on VitekMS to make sure there was not a problem with the media since there was growth on the plates, and the growth resembled *B. velezensis* and not *B. cereus* on the selective media. No further workup was done when the ID came off as *B. velezensis*. Whole genomic sequencing was not done since there was no clinical isolate from the ill individuals to compare to the food isolate.

## RESULTS

**Fig 3:**
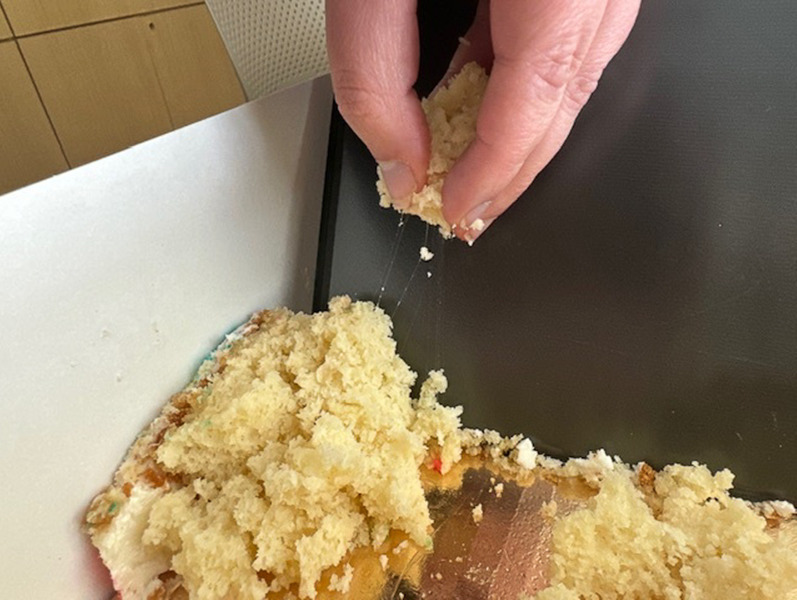
Strings (ropes) in sample of the cake from the outbreak of gastrointestinal illness. Photoshop 26.2.0 was used to upscale the image for publication.

Thirty-five survey responses were collected from 16 May through 15 June 2024, with the majority of responses collected by 20 May. The examination of the directions on the cake mix package found this statement: “Cake Mix is not ready-to-eat and must be thoroughly cooked before eating. To prevent illness from naturally occurring bacteria in wheat flour, do not eat raw batter; wash hands and surfaces after handling” (verbatim from package). The macroscopic examination of the cakes was done in the field (see Fig. 3). The strings (ropes) are clearly visible when the cake is torn apart.

In the retrospective review of the inspection reports of the bakery, inspections occurred approximately 10 and 3 months before outbreak onset. There had been two major issues: the handwashing sink and the refrigerator were not working properly. TCS foods are required to be held at 41°F or below. If they are above 41°F for than 4 hours they must be discarded. In addition, the sink’s hot water (100°F) and cold water must be supplied for employee handwashing use. The sink had been out of order 5 months prior to the outbreak onset. The owner had stated that they do not use real cream cheese in the icing or filling, though these ingredients were subsequently found. A review of the bakery operations found the baking process consisted of setting the oven to 325°F, coating the bottom of the pans with non-stick cooking spray, mixing the cake mix, oil, and water, pouring the batter into the pans, and cooking for 25 to 30 minutes. Cooking times vary by cake size. The two ovens used include a full-size convection oven capable of holding five full-size pans at 18″ by 26″. The second oven is half the size. There were 9 half-sheet cakes, 11 quarter sheet cakes, and 10 round cakes. These three batches would have taken at least 2/3 of each 50 lb bag of cake mix. The mixing process, if done efficiently according to the manufacturer’s directions, would have taken at least 15 minutes (mix and water partially poured in, mixed for 3 minutes, scraped down, more water added, mixed until smooth for 1 minute, mix another 2 minutes, scraped down, remaining oil and water added in at slow speed and mixed for another 2 minutes), with another 15 minutes of pour time and pan preparation. Moving the battered pans into the oven would have taken another 10 minutes. This rough estimate provides 40 minutes for one of three cooking batches. If the cakes were split up between the two ovens to allow for proper convection around the pans, the next pulse of mixing and pouring would start during the baking of the first batch. The mixer could be cleaned or used for the same ingredients. During the second pulse, the mixing and pouring would have to stop to take the cakes of the first pulse out of the oven for cooling. These cakes would go onto the cooling racks. The mixing and pouring of the second pulse would begin again, and once completed, these cakes would go into the ovens. The third pulse would start while the cooking of the second pulse was underway. The third pulse would have to be halted to take the cakes of the second pulse out of the oven and to take the cakes from the first pulse from the cooling racks and wrap them in plastic wrap and move the first pulse cakes that are wrapped in plastic wrap on the bakery racks overnight to be decorated the next day. The pouring and mixing of the third pulse would restart, and the process possibly halted again to take the second pulse cakes to the cooling racks. The pouring and mixing for the third batch would be completed and placed in the ovens. The second batch would be taken off the cooling racks and wrapped in plastic and placed on the bakery racks overnight. The third batch would be taken out of the ovens and placed on the cooling racks. Once cooled for 1 to 2 hours, these cakes would be wrapped in plastic wrap and placed on the bakery racks overnight to be decorated the next day.

As part of the FMEA process and literature review, a table was constructed of risk factors for spoilage related to baked cakes (Table 1) (17, 19, 23). Bread is considered shelf stable even if it has a_w_ as high as 0.92 and has been baked at high temperatures, provided the package has not been opened. There have been well-documented outbreaks from raw flour. It is common knowledge that raw flour is not “a ready-to-eat food.”

**TABLE 1:** Cake has lower acidity and higher water activity conducive to rope spoilage^*a*^

Growth factor	Acidity (pH)	Water activity (a_w_)
Rope spoilage low	<4.6	<0.6
Rope spoilage intermediate	>4.5	0.6–8.85
Rope spoilage high	>4.5	>0.85
Bread	5.1–5.4	0.95–0.97
Cake	6.7–8.0	0.81–0.90
*Bacillus velezensis*	5–10	0.90–1.0
*Bacillus subtilis*	4.8–9.2	0.929

^*a*^ Data are from references 17 and 20. Note that other authors have found similar ranges for strains of *B. velezensis.*

**Fig 4:**
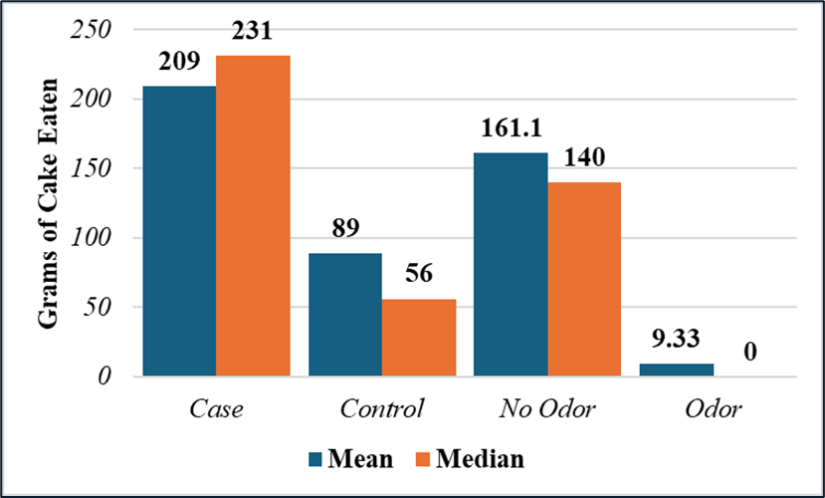
Cases ate over twice as much cake as controls (independent t test of means 209 g versus 89 g, *P* = 0.011 and Wilcoxon Rank Sum test *P* = 0.014). Persons who experienced a bad odor ate little or no cake (independent t test, equal variances not assumed, *P* < 0.001).

**Fig 5:**
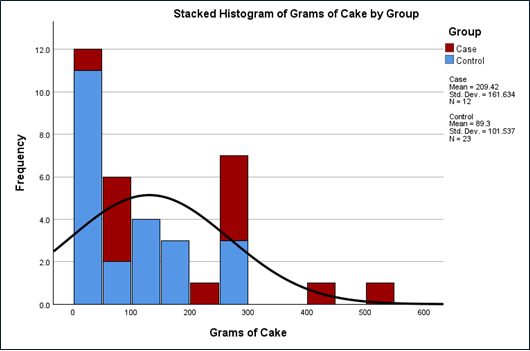
Cases had more grams of cake than controls.

**Fig 6:**
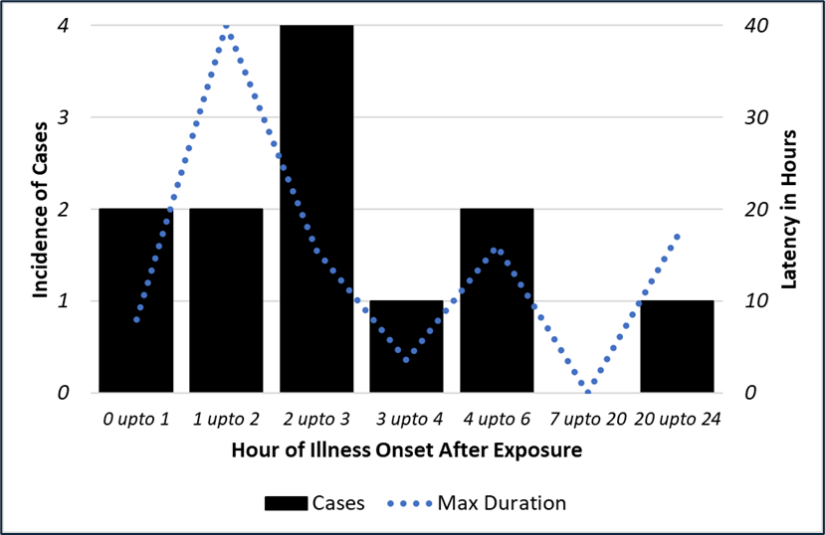
Symptoms were consistent with spore-forming bacteria, including initial stomachache, cramps, and/or nausea followed by latent diarrhea.

The FMEA process identified a list of potential factors contributing to bacterial proliferation, including increased spore loading during the agricultural process (either from increased moisture from grain grown in a wetter environment, moisture exposure during storage, or added as an inoculant during farming), high pH due to lack of acidity regulators, leaving batter on the mixer for extended periods, shortened cooking times (limited kill step and increased a_w_), incomplete cleaning of surfaces and pans with acidic cleaner, long cooling times (e.g., pans stacked too close together for proper cooling to occur), baked goods wrapped when warm, too much a_w_ in the final product, and the latency (i.e., incubation time between baking and exposure) after icing the cakes before being ingested, consistent with growth curves for *Bacillus* as synergistic contributors to the outbreak. Consistent with the FMEA findings, there was a significant difference between those who experienced odor and ate an average of 9.3 g versus 161 g among those that did not experience odor (Fig. 4) and the difference in grams of cake eaten by the cases versus controls. Fig. 5 shows the histogram of exposure to cake by case-control status. The odds ratio of illness if exposure to cake was 9.23 (1.02–83 95% CI, Chi-square *P* value = 0.025, Fisher exact *P* = 0.027), Table 2. Linear regression found that the presence of odor decreased the number of grams of cake eaten by 151 g (95% CI 103–205 g, *P* = 0.001). The presence of ropes or other macroscopic signs of spoilage were not significant predictors. Table 3 shows the line list of subjects. Fig. 6 shows the outbreak curve of incubation and duration in hours. The cakes were picked up by the customer and distributed to 17 locations (14 weekend staffed locations and 3 unstaffed). Cakes not eaten included 3 staffed and 3 unstaffed areas, for a total of 11 departments exposed. The symptoms started to develop 25 minutes to 4.5 hours after eating cake. All the cases except one had acute enteric symptoms, 5 of the 12 cases had latent diarrhea (one person only had latent diarrhea about a day later). The exposures started around 14:00 hours on 12 May, with the earliest onset around 14:30 and the latest onset of acute symptoms around 19:00 that day.

**TABLE 2:** Exposure to cake is associated with illness over ninefold (odds ratio 9.23 [1.02–83.3 95% CI, *P* = 0.02701])

	Sick	Not sick
Exposed	12	13
Not exposed	1	10

**TABLE 3:** Subject list of exposure, symptoms, and observations

Grams of cake eaten	Incubation hours	Duration hours	Ropes	Odor	Stomachache	Nausea	Cramps	Diarrhea	Other observations/symptoms
0	0	0	0	0	0	0	0	0	
0	0	0	0	0	0	0	0	0	
0	0	0	0	0	0	0	0	0	
0	0	0	0	0	0	0	0	0	
0	0	0	1	1	0	0	0	0	
0	0	0	1	1	0	0	0	0	
0	0	0	1	1	0	0	0	0	
0	0	0	1	1	0	0	0	0	Discoloration
0	0	0	1	1	0	0	0	0	Fluffy
14	0	0	1	0	0	0	0	0	Very dense moist
56	0	0	0	0	0	0	0	0	
84	0	0	0	0	0	0	0	0	
112	0	0	1	0	0	0	0	0	
140	0	0	0	0	0	0	0	0	
140	0	0	0	0	0	0	0	0	
140	0	0	0	0	0	0	0	0	
168	0	0	0	0	0	0	0	0	
168	0	0	1	0	0	0	0	0	
192	0	0	0	0	0	0	0	0	
280	0	0	0	0	0	0	0	0	
280	0	0	0	0	0	0	0	0	
280	0	0	1	0	0	0	0	0	
7	2.5	15.0	1	0	0	1	0	0	Headache
56	2.5	14.0	0	0	1	1	0	0	Taste off
56	3.0	3.5	1	1	0	1	0	0	
70	1.2	40.3	1	0	0	1	0	1	Heartburn
70	3.5	14.0	1	0	1	0	0	0	
210	0.9	1.4	0	0	0	1	0	0	Undercooked doughy
252	1.5	3.0	1	0	0	1	0	0	
280	2.0	15.5	0	0	0	0	1	1	
280	2.5	1.0	1	0	0	0	1	0	
280	4.5	16.0	0	0	1	1	1	1	
448	21.0	17.0	1	0	0	0	0	1	
504	0.4	8.0	0	0	1	1	0	1	

## DISCUSSION

The authors believe the prevalence of *B. velezensis* as the cause of foodborne outbreaks is underreported for a variety of reasons. We found that *B. velezensis* was opportunistic due to the proliferation factors that are present. The current outbreak, as well as the identification of the primary pathogen *B. velezensis,* would not have been recognized if it were not part of a larger network celebratory event. Given the emergent recognition of the outbreak-causing scenario and the mild illnesses, most persons would not have linked their illness to any particular event… unless sampled retrospectively for symptoms across a variety of sites across multiple adjacent cities. While the sample size was sufficient to determine a statistically significant association across a variety of methods and to identify a dose-response relationship between grams of cake eaten and symptoms, the number of subjects was small. This may affect the generalizability of the results, particularly to other types of baked goods with lower levels of nutrient-rich ingredients. As a counter to this critique, multiple persons from 11 different sites were sampled across adjacent cities. The authors would use “opportunistic” and “primary” to describe the pathogen. *B. velezensis* did cause illness (albeit mild and self-limiting) in persons actively employed. Therefore, we cannot say it is only an opportunistic pathogen. It may require a large dose to cause illness, and a dose-ascending challenge could be the subject of future investigation. The incidence of outbreaks involving subclinical illnesses from *B. velezensis* is an area that should be studied further.

The presence of spoilage provided some early warning signs to the potential cake eaters. This outbreak meets all the original criteria that Bradford Hill set to prove causation (24). The use of *B. velezensis* is preferable over the use of chemical fungicides for agricultural purposes for several reasons, including the avoidance of the negative effects on the environment and human health from exposure to chemicals, and the development of resistance to fungicides (25). The bacteria appear to create a symbiotic relationship with plants by producing secondary metabolites that inhibit fungi and/or boost the plant’s fitness. This supports that *B. velezensis* is an organism used in the agricultural grain growing process and is a natural biocontrol agent that can be added at various stages and quantities during that process. The characteristic of this family of bacteria is the formation of endospores. These endospores, even at a small amount, could survive the manufacturing process of flour used in baking. Nutrient and non-nutrient substances can cause *Bacillus* spores to germinate. Nutrient germinant substances in baked goods include amino acids, sugars, and nucleosides. Non-nutrient germinant includes cationic surfactants (surfactants are substances like soaps or detergents). Spore germination can occur with a nutrient or non-nutrient germinant. It is logical that as the *Bacillus* species produce lipopeptides, once germination starts and lipopeptide production begins, the surfactant and antimicrobial properties of the secondary metabolites would initiate an exponential growth rate in the presence of enough nutrients, and other potential competing bacteria would be inhibited. The residual chemicals left behind during use of detergents to clean utensils, mixers, and bakery pans could also contribute to germination of spores in the baked goods, especially if the cakes were undercooked. Reports from those affected include observations of “undercooked” and “very dense-moist.” These appear to be consistent with cakes that were undercooked. The presence of “ropes” indicates the presence of oligopeptides produced by *B. velezensis* during its growth phase. The smell that was reported is consistent with the production of secondary sulfur metabolites that are known to be produced by *B. velezensis*. The absence of any other organisms is consistent with the antimicrobial effects of *B. velezensis’* production of a microorganism inhibiting secondary metabolites as well as the baking process. The significant odds of illness if exposed to cake, the statistically significant *protective* impact of the bad odor, and the dichotomous presentation of near onset and latent diarrhea is further proof of causation of illness from a spore-forming bacteria like *B. velezensis*. The positive culture of *B. velezensis* further supports proof of causation that the *B. velezensis* caused an outbreak of illness characterized by classic symptoms of baked goods spoilage known as “ropey spoilage” including stomachache, nausea, cramps, and diarrhea among those who ate cakes during the celebration. The small retail bakery had a comparatively large order, and to increase throughput, it is conceivable that higher heat and less baking time were used per cake. This would explain the observations that cakes were dense *and* moist in the middle. The review of past inspections found baking pans had considerable buildup, suggesting cleaning of the pans had not been a high priority. Perhaps the bakers thought high temperature and time would destroy any bacteria. The persons who observed warning signs and did not eat the cake were not sick; those who ate the cake, with or without warning signs, had a statistically significant chance of becoming sick with an illness consistent with that of spore-forming bacteria. There is also a dose-response relationship between the amount of cake eaten and symptoms. Even when the presence of odor by potential cake eaters warned of spoilage, the exposure to cake eating was still significant in this small sample size.

Proper handwashing is one of the most effective and important steps in the prevention of food contamination and possible foodborne outbreaks. The two critical violations could have created possible food safety/sanitation concerns. Was the staff employing proper handwashing techniques even though there had been, at least historically, water temperature issues? At one point, it was noted that the hand sink needed to be properly supplied. Having a refrigeration unit that was unable to maintain TCS foods at 41°F or less is a critical factor. If the food was out of temperature for an extended time, that will certainly permit the growth of potential pathogenic organisms. That could be another contributing factor toward a potential foodborne incident.

The predominant conundrum remains, though, did these two ongoing critical violations cause, or at the very least, contribute to the ropey cake issue? Based on the historical review of the inspection reports, the health inspectors believed they did not have sufficient evidence to draw a conclusion that handwashing, food buildup on pans, or lack of refrigeration led to a condition that contributed to the ropey cake issue. This was not because those factors are not potential contributors to food safety, but that there was no evidence that these factors were thought to be in play by the health inspectors during this outbreak.

Once the batter was made, how long did it sit out before it was poured into the cake pans? There are no documented historical violation notations to suggest that such a practice was ever an issue. Another point of concern is whether these cake products should be considered TCS foods. Dry baked goods without perishable toppings/fillings are non-TCS. Dry flour is also considered to be non-TCS until it is exposed to moisture. Bakers typically like their cakes to be moist to some degree, what is the a_w_ of these products? At what point does the a_w_ level become sufficiently high enough that such cakes could be considered TCS? These cakes were never tested for the a_w_ within the final product. It is known that undercooking cakes is a predominant risk factor for cake spoilage. Did the ropey cake issue originate from something that occurred at the processing plant from where the cake mix was purchased by the bakery? It is unlikely that an unusually large number of spores in the cake mix would have caused an outbreak of illness if the proper acidic pH was maintained, and if cooking temperature and cooking time decreased the a_w_ to below 0.85. While there are some concerning violation trends identified within the historical inspection report data, it does not provide “smoking gun” evidence needed to conclusively state that this was the result of something the bakery did during the production of these cakes. The classic symptoms of cake spoilage, the incubation and duration of symptoms, and the dose-response relation are considered proof of causation using standard Bradford Hill criteria.

For further scientific proof that the baking process was at fault, let’s consider the growth curve of *B. velezensis*. There is a widespread agricultural practice of applying *B. velezensis* to seeds, soil, and/or the base of grain crops (26). Indeed, the endospores of the bacteria could survive the cooking process, and from the FMEA, a shortened cooking time, along with a prolonged incubation time (the time from baking to ingestion), was identified as the critical steps from the field to final exposure.

**Fig 7:**
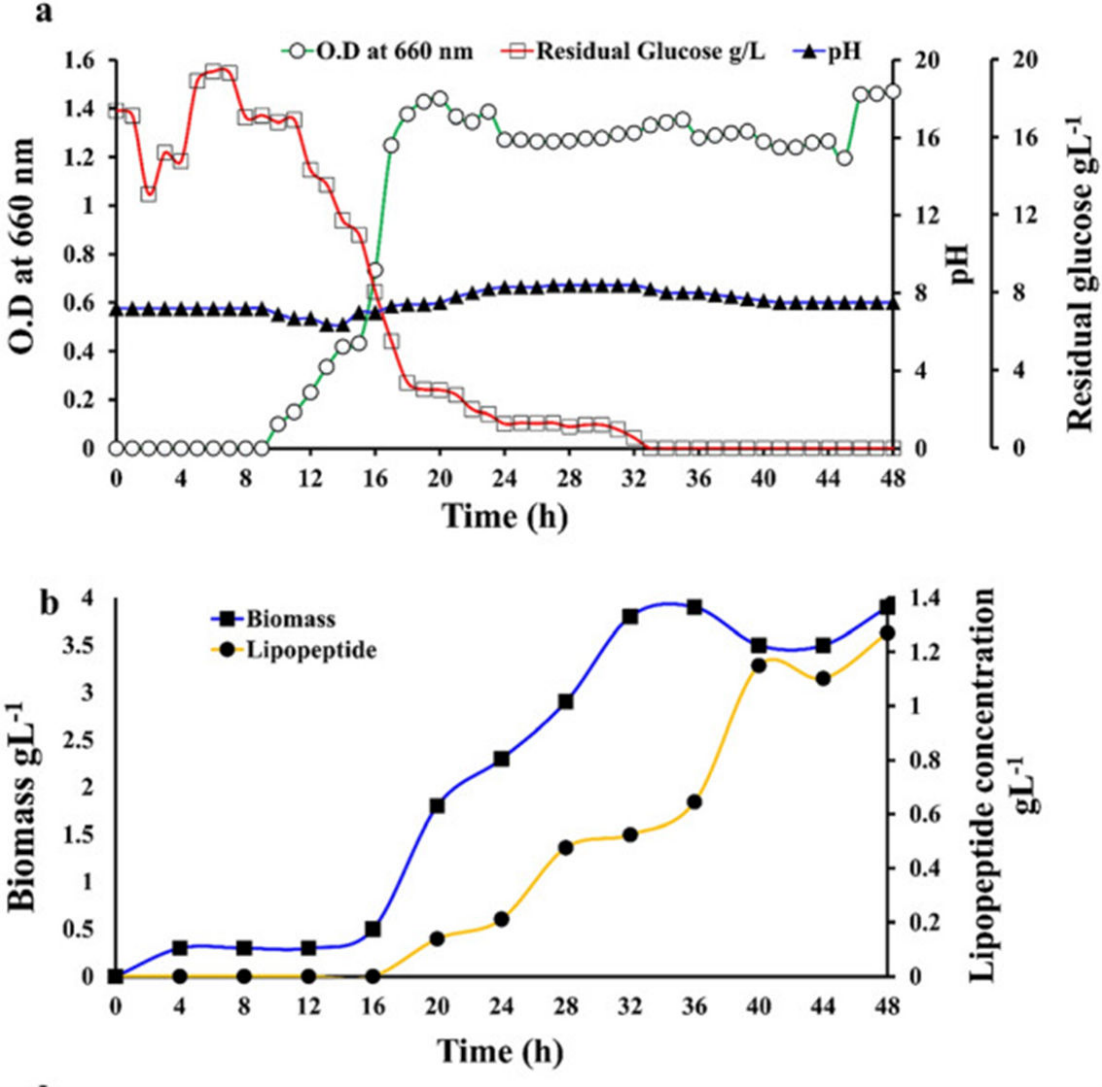
*B. velezensis* growth curve is consistent with the presence of lipopeptides and the switch to fermentation that would produce signs of spoilage, including the presence of lipopeptide strings and sweet rotting pineapple smell. (Republished from *AMB Express* [27].)

The bacterial growth curves suggest that oxygen is utilized up through about 20 hours as the bacterial biomass increases, and the biomass starts to level off at around 32 hours with an increased production of lipopeptides after 36 hours (see Fig. 7 by Barale et al.) (27). The growth curve of *B. velezensis* and production of secondary metabolites is biologically consistent with the strings observed in the cake. Also consistent are other signs of spoilage, such as the smell of sugar alcohol (the sweet pineapple smell reported) as anaerobic production seems to occur as early as hour 16. These classic signs of spoilage have been studied for over 100 years (19). “Ropiness is associated with a patchy discoloration and a stringy bread crumb and characterized by an unpleasant sweetish odor resembling rotting melons or pineapples that is caused by the release of volatile compounds including diacetyl, acetoin, acetaldehyde, and isovaleraldehyde” (17).

While the estimation of bacterial counts is theoretically possible, it is not a routine practice for determining the dose-loading per cake eaten. Given that the batter was mixed, it is reasonable to assume that *B. velezensis* would be uniformly distributed throughout the batter. The cooking process would destroy spores and any vegetative bacteria on the outer portions of the baked cakes. The size of the cakes, cooking times, and post-baking variations, such as cooling times and environmental factors, could impact the bacterial load. Additionally, the susceptibility of individuals to the bacteria and the amount of cake consumed are significant factors. We controlled for the dose by estimating the amount of cake each person ate, and these estimates were statistically significant using both parametric and non-parametric methods. Regarding the critique about the lack of isolation and comparison of *B. velezensis* abundance, these steps were not feasible within the scope of our study. As stated earlier, no clinical isolates were available to compare to the food isolate, so genomic sequencing was not done. However, the uniform distribution assumption, the visual observation by those affected, and the statistically significant modeling for the amount of cake consumed causing illness provide proof of causation that the outbreak of illness was caused by *B. velezensis* that was in the ingested cakes.

The most reasonable explanation for the presence of the strings, the sweet rotting pineapple smell, and the statistically significant presence of symptoms among those exposed in a dose-dependent manner is consistent with the scenario of shortened cooking time and handling post-baking, before ingestion. The composition of an undercooked cake mix containing amino acids and glucose would be a nice medium for spore-forming bacteria. In the retrospective review of the bakery’s past inspection reports, it did not provide a smoking gun related to this outbreak. What did those inspection reports provide? A history of delayed repairs suggests a bakery with limited resources to fulfill a large order. The small bakery had two ovens and one cooking rack. The overlapping processes of mixing, pouring, baking, cooling, and wrapping of at least three or more batches of cakes would have kept the bakery staff very busy, and further supports a breakdown in following the exacting bakery processes required to maintain baked items as a non-TCS food item. Any combination of factors including the lack of pH regulation, shortened cooking times (limited kill step and increased a_w_), incomplete cleaning of surfaces and pans with acidic cleaner, long cooling times (e.g., pans stacked too close together for proper cooling to occur), baked goods wrapped when warm, too much a_w_ in the final product, and incubation time consistent with growth curves for *Bacillus* species would lead to spoilage presenting as smelly, ropey cake. Given the 72 hour delayed ingestion of cakes, cakes that were moist-dense and doughy consistent with undercooked cakes, ingestion of cakes was followed by dose-dependent mild enteric illness consistent with some strains of *B. velezensis*, latent diarrhea consistent with spore-forming bacteria of the genus *Bacillus*, strings consistent with secondary metabolites and biofilm production of *Bacillus* species, an overlapping-complex bakery process, and cakes positive culture for *B. velezensis*, it is concluded that *B. velezensis* is the cause of this outbreak. Due to improper bakery cake making, specifically not cooked for the proper length of time, and the prolonged storage of at least 72 hours, led to the proliferation of *B. velezensis*. The most probable sources of the bacteria are naturally occurring spores in flour, as *B. velezensis* is not only ubiquitous in the environment but also a well-known inoculant of grain seed and grain products used to promote growth and prevent wheat blight during the agricultural process. The continued use of the mixer without cleaning over multiple batches allowed for bacterial proliferation, altered cooking duration, and inadequate temperature insufficient to kill germinated bacteria, inadequate cooling before wrapping allowed moisture building that enabled temperatures to reach optimal levels for an extended period of time for *Bacillus* to grow, improper environmental conditions for storage by the customer before serving allowed for further growth of *Bacillus*, and prolonged latency to ingestion after cooking are single or combinations of causes that are biologically feasible causes of this outbreak. Biological feasibility contributes further to the proof of causation overall.

Recommendations for controlling disease and/or preventing/mitigating exposure—the FDA has been understaffed and underfunded, putting consumers at risk for food and drug safety (28). From 2018 to 2022, the number of inspectors declined by 20% due to low pay and significant travel. FDA officials say the agency needs to double its workforce to meet inspection demands. The food industry’s major complaint about the FDA is slow response to crisis and overemphasis on pharmaceutical inspectors with little or no food industry experience (29). Food safety experts have long complained that the FDA’s food oversight arm is understaffed and underfunded. Some say the agency has also prioritized the drug and medicine side, often hiring leaders with medical backgrounds and little food industry knowledge (30). If the war in Ukraine disrupted grain supply to the United States, the issues with the FDA to assure the safety of imported grain could be an issue. However, there are four reasons the issues with the U.S. FDA are not related to this potential re-emerging ropey spoilage of grain baked foods. The first reason is that almost all countries in the world were affected. Georgia, Armenia, Kazakhstan, Azerbaijan, and Mongolia were the most vulnerable (31). The U.S. is not among countries that rely heavily on food imports. The second reason is that Sabillon and Bianchin have suggested that the wheat grown in wetter environments carry higher microbial loads (32). This is a growing concern because of climate change. We don’t think it is an issue because in the U.S., grain elevator operators pay for grain that has the right amount of moisture. Too much moisture, too much weight, and too much cost. The third reason we don’t believe the FDA issues let a batch of bad flour on the market is that some strains of *B. velezensis* are approved as probiotics and are safe to be ingested orally (33). The agricultural application of endospores provides for increased crop production and a natural biocontrol agent. It is not known what amount of *B. velezensis* on endospores in “raw flour used as an ingredient” in powdered ready-to-use cake mix is harmful or beneficial. The presence of *B. velezensis* could have prevented a worse outbreak because of their antimicrobial secondary metabolites that presumably caused the acute but mild enteric symptoms. Without further study, it is unknown if the manufacturer’s directions should be updated to prevent this type of outbreak from occurring again. This does present the possibility of an unintended consequence, that more spores entering the food production chain could lead to more spoilage. The fourth reason is that we believe if the proper acidic pH was maintained, and if cooking temperature and cooking time decreased the a_w_ to below 0.85, that even many spores in the cake mix would *not* have led to enteric illness. The potential for continued use of the mixer allowing for bacterial proliferation does not seem to be an important risk factor for this outbreak. Bailey and colleagues found that pre-baking equipment contributed little to the total count of colony-forming units in final products (34).

This outbreak appears to be the first mention of *B. velezensis* as a cause of a foodborne outbreak. Updates to the *Bacillus* taxonomy starting in 1973 which separated species from *B. subtilis* by integrating *B. amyloliquefaciens* subsp. plantarum and *B. methylotrophicus* into *B. velezensis* as of 2017 (35). This accounts for why *B. subtilis*, *B. cereus*, and other *Bacillus* strains are listed as common causes (17, 20), but without mention of *B. velezensis* listed as a cause of rope spoilage. With our report, *B. velezensis* can now be reported as a cause of rope spoilage. *Bacillus* species are spore-forming bacteria. The persistence of *Bacillus* spores in the environment was highlighted by the largest bioterrorism attack in the history of the United States (2). Aerosolization of spores from *Bacillus* species has been presented by a variety of researchers related to the evaluation of the contamination of the Hart Senate building from one of the anthrax attacks after 9/11 (36). Of interest is the extremely low dose of just one to three endospores to cause disease. The evaluation of the Hart Senate building clearly demonstrated “a potential for secondary aerosolization” of viable spores originating from contaminated surfaces of an indoor environment. In a survey of the microbiology of wheat and flour milling, *Bacillus* species were the most frequently detected in the survey (37). Furthermore, those researchers found that the quality of incoming wheat has a strong influence on the quality of the end “raw” product. This is consistent with our supposition that there may have been some upstream factors that impacted the quality of the product before baking, though we believe those upstream factors would be minor as related to the other factors more proximal to the “baked” end product.

**Fig 8:**
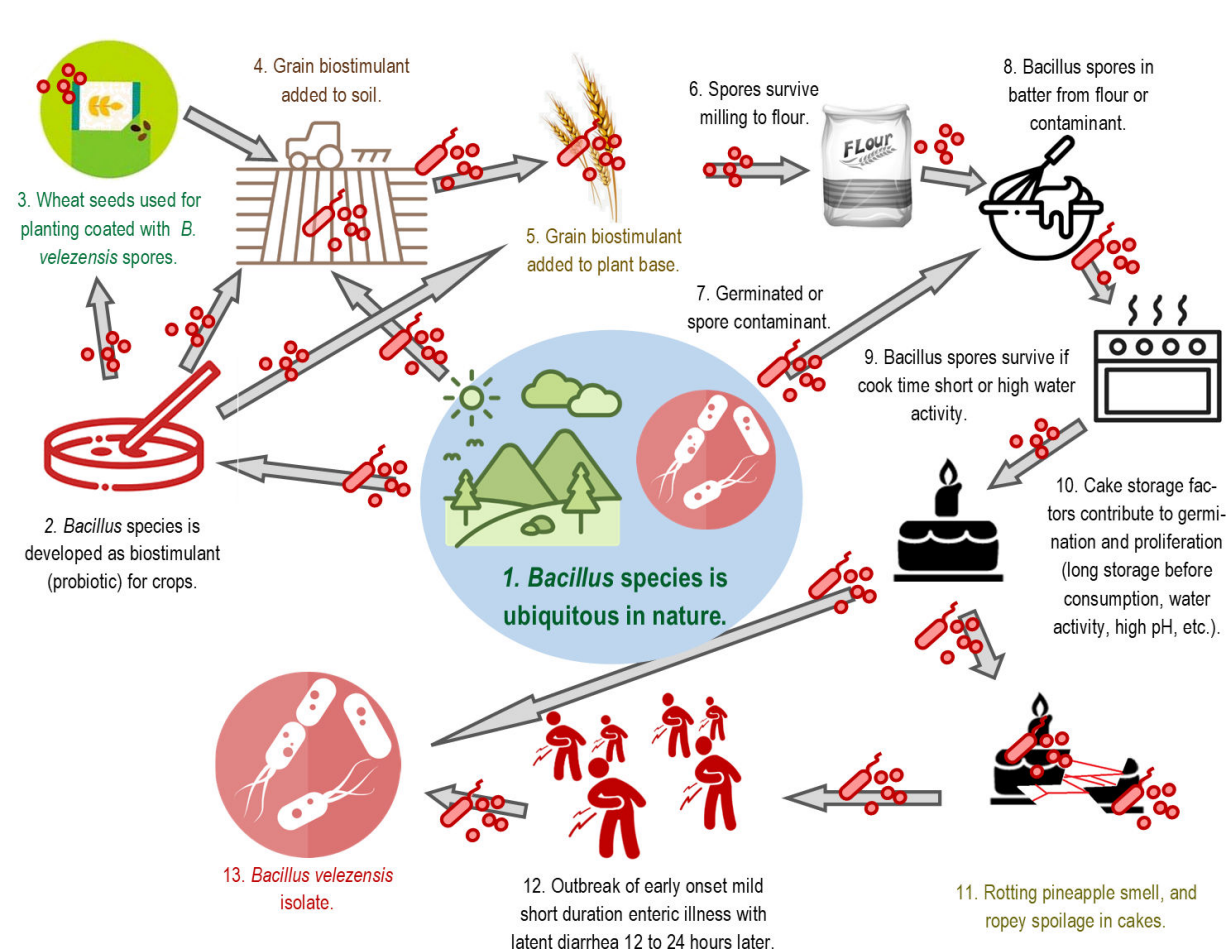
Biological feasibility of introduction of the causative agent in the food chain and given ample incubation time causing the first reported outbreak of illness caused by *Bacillus velezensis*.

In summary, this outbreak was caused by the proliferation of *B. velezensis* in the 40 cakes that were made in one work period, frosted the next day, and had ample incubation time given the known growth curves and growth conditions (Fig. 8). In the context of food production, extrinsic environments refer to external factors and conditions that can influence the process but are not directly controlled or manipulated. These environments can affect the quality and safety of the food product. The inoculation of the product may occur from the extrinsic environment. Controlled environments, on the other hand, are those where conditions are carefully monitored and managed to ensure specific outcomes, such as lab-produced bacteria or the conditions within the finished product after baking.

The authors acknowledge the low bacteria count on the wheat endosperm. Given that the initial milling process includes the bran, germ, and endosperm, it is inevitable that naturally occurring *Bacillus* (vegetative and endospores) and *B. velezensis* biocontrol strain endospores applied in the field become ubiquitous throughout the mill and milling process and in the final processed flour.

Figure 8 suggests that there is a potential exposure pathway and the biocontrol strain on wheat is a possible source. Exploring those pathways would take an industry-wide survey at every stage of production. The survey would have to account for strains of grain, strains of *Bacillus*, variations in production methods, and variations in storage, transport, milling, etc. There are mediating factors that can cause an outbreak to occur, including extrinsic environmental conditions (37). These factors would be assignable causes for an outbreak and would otherwise be hard to detect retrospectively. In the unique scenario as in this outbreak where baked goods were prepared simultaneously from the same source ingredients, stored in a similar fashion, and inadvertently allowed to have the necessary incubation time before ingestion, would the signs of spoilage be recognized and linked to the symptoms of exposed persons at various sites across the nearby adjacent cities?

Higher than the recommended amounts of biocontrol agent applied in the field is only one of many possible exposure pathways. It is a possibility that standard levels could be applied, and given the incubation period after suboptimal baking, inadequate environmental factors during storage of the final product, and longer than normal latency before ingestion of the bacteria and/or spores may cause some persons to have enteric symptoms.

This appears to be the first reported outbreak of illness caused by *B. velezensis*. We were unable to identify in the literature that there were no specific instances of *B. velezensis* as a cause of the re-emergence of rope spoilage in the bakery industry. It is concluded that the dry cake mixes and overall baking methods by the industry enabled the finished cakes’ subsequent variability in the environmental conditions, which led to further bacterial proliferation. Proper storage of ingredients, equipment cleaning with acidic cleaners, acidic batter pH, correct cooking times, correct cooling procedures, and proper environmental conditions of storage of the product are recommendations to the bakery to maintain proper shelf life of finished cakes. The need to avoid resistance among pathogenic organisms will require the continued use of agents like *B. velezensis* in the agroindustry. Given the ability of *B. velezensis* to grow at similar pH levels and water activity as many baked goods, there is a need for research to study issues along the entire food chain. The impact of biocontrol additives on food production and food safety needs to be balanced. Further study is required to determine if biocontrol additives would be likely to control spoilage in cakes. This includes further study of the virulence of *B. velezensis*. All the illnesses in this outbreak were mild. In this outbreak, there were no deaths and no hospitalizations associated with exposure to *B. velezensis*. In conclusion, this outbreak of mild enteric illness was caused by *B. velezensis*.
